# Comparative Analysis of Fruit Metabolome Using Widely Targeted Metabolomics Reveals Nutritional Characteristics of Different *Rosa roxburghii* Genotypes

**DOI:** 10.3390/foods11060850

**Published:** 2022-03-17

**Authors:** Lanlan Jiang, Min Lu, Tianzhi Rao, Zeyang Liu, Xiaomao Wu, Huaming An

**Affiliations:** Guizhou Engineering Research Center, Fruit Crops/Agricultural College, Guizhou University, Guiyang 550025, China; j17385313153@163.com (L.J.); mlv@gzu.edu.cn (M.L.); R1786970809@163.com (T.R.); lzy0104040856@163.com (Z.L.); wuxm827@126.com (X.W.)

**Keywords:** *Rosa roxburghii* Tratt., *Rosa roxburghii f. eseiosa* Ku, widely targeted metabolomics, characteristic metabolites, bioactive substances

## Abstract

The fruits of *Rosa roxburghii* (*R. roxburghii*) Tratt., which are rich in bioactive compounds, provide numerous health benefits, yet the overall metabolism of *R. roxburghii* fruits and the metabolic profiles among different genotypes of *R. roxburghii* fruits are not fully understood. In the research, we used ultra-performance liquid chromatography/tandem mass spectrometry analysis to identify and quantify metabolites including phenolic acids, amino acids, and organic acids in six *R. roxburghii* genotypes; a total of 723 metabolites were identified. Comparative analysis showed some different characteristic metabolites in each genotype. Moreover, flavonoids, triterpenoids, and phenolic acids were significantly correlated with the antioxidant capacity of the fruit extract. Our results suggest that *R. roxburghii* fruits have rich bioactive metabolites beneficial to human health and that Rr-7 and Rr-f have more potential to be used as medicinal material or functional food than other genotypes. This research provides helpful information for developing new functional foods of *R. roxburghii* genotypes.

## 1. Introduction

*Rosa roxburghii* Tratt. is a traditional fruit tree growing in China that is rich in bioactive compounds, including L-ascorbic acid [1], amino acids [2], flavonoids [3], triterpenes [4], and phenolic compounds that provide numerous health benefits [5,6,7]. Since the medicinal value of *R. roxburghii* has been discovered, phytochemical studies have become more popular. The components and content of organic acids and ascorbic acid of *R. roxburghii* were analyzed by liquid chromatography, respectively [1]. Eighteen amino acids were detected in *R. roxburghii* [8]. In addition, the GC-MS method was performed on *R. roxburghii* fruits and 44 volatile compounds were identified [9]. Meanwhile, Yang et al., identified 30 phytochemical compounds in the aqueous ethanolic extract of *R. roxburghii* fruits by UPLC-QTOF-MS and used the GC-MS method to analyze the active products of the volatile oil of the *R. roxburghii* fruit, identifying 78 kinds of chemicals in active products, among which flax oil acid has the highest content [10]. *R. roxburghii* has shown beneficial effect against oxidative stress and has been suggested for the treatment of hypoglycemic activities, ovarian cancer, antimutagenic, antioxidant, vitamin C deficiency, etc., [6,7,11,12]. The water-soluble extract of *R. roxburghii* fruit can significantly reduce the weight gain and serum and liver lipid levels of hyperlipidemia mice [13]. These beneficial functions are closely related to the chemical composition of the plant. *R. roxburghii* is also widely used in the food industry. To date, *R. roxburghii* has been used for the productions of fruit juice, dried fruit, fruit wine, and fruit cake. To sum up, these studies have focused on specific metabolite categories of *R. roxburghii* such as ascorbic acid [1], amino acids [8], and flavonoids [3]. Yet, the overall metabolism of *R. roxburghii* fruits and the metabolic profiles among different genotypes of *R. roxburghii* fruits are not fully understood.

Over the years, researchers have collected a large number of germplasm resources and obtained certain lines with excellent characters [14], some of which have been approved, including ‘Guinong 1′ (Rr-1), ‘Guinong 5′ (Rr-5), and ‘Guinong 7′ (Rr-7) [3,15]. At present, the main planting area of *R. roxburghii* in Guizhou Province reaches 133,300 hectares, with Rr-5 representing the most cultivated variety. In addition, some genotypes with potentially good quality were found; for example, the fruit size of Rr-3 and Rr-4 are larger than those of other *R. roxburghii* germplasm resources and could be a candidate for high-yield varieties in the future, while the fruit of *R. roxburghii f. eseiosa* (Rr-f) is smooth and prickleless, suitable for fresh food. The three *R. roxburghii* genotype fruits have their own characteristics and are favored by producers and consumers, therefore they have great production potential. Moreover, different genotypes of *R. roxburghii* are rich in different bioactive substances and their nutritional value and medicinal value are also different. Therefore, we use widely targeted metabonomics to explore the metabolic profiles of various *R. roxburghii* genotypes in this study.

The ultra-performance liquid chromatography/tandem mass spectrometry (UPLC-MS/MS) enables accurate qualitative quantification of metabolites. As a source of nutrition and energy, plants can synthesize a great deal of metabolic substances with different biological functions. Therefore, the studies of metabolomics have a great significance in improving crop yield and enhancing food quality. At present, there are applications in crops such as wild rice [16] and buckwheat [17]. The main objective of the metabolomic analysis is to detect and screen metabolites with momentous biological significance and statistical differences from biological samples and to expound the metabolic processes and mechanisms of change of organisms on this basis. To better understand the differences of *R. roxburghii* genotypes, in this study we used UPLC-MS/MS analysis to identify and quantify metabolites including phenolic acids, amino acids, and organic acids in six *R. roxburghii* genotypes. We examined fruit quality, bioactive substance content, antioxidant capacity, correlation analysis, and widely targeted metabolomics analysis on six *R. roxburghii* genotypes fruits to find the characteristic metabolites and biomarkers of different genotypes as well as the main differential metabolic pathways. The research can provide the theoretical basis for nutritional value assessment and food development and can help identify the best genotypes according to their purpose in the food processing industry, medicine, plant breeding programs, and other aspects.

## 2. Materials and Methods

### 2.1. Plant Material

**Figure 1:**
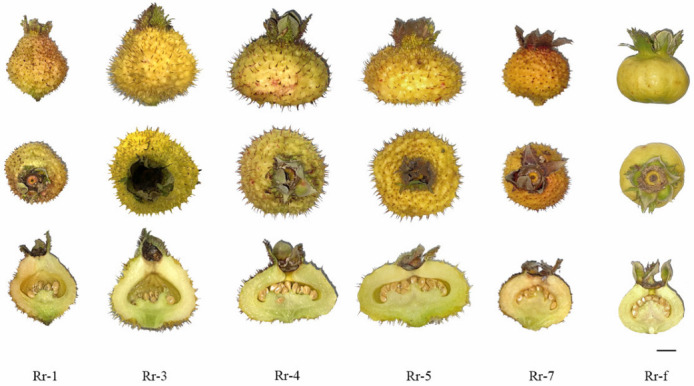
Mature fruits of 6 *R. roxburghii* genotypes. Scale bar = 1 cm.

Eighteen biological samples of 6 *R. roxburghii* genotypes, including Rr-1 (*R. roxburghii* Tratt. cv ‘Guinong 1’), Rr-3 (*R. roxburghii* Tratt. accession ‘Guinong 3’), Rr-4 (*R. roxburghii* Tratt. accession ‘Guinong 4’), Rr-5 (*R. roxburghii* Tratt. cv ‘Guinong 5’), Rr-7 (*R. roxburghii* Tratt. cv ‘Guinong 7’), and the variant Rr-f (*R. roxburghii f. eseiosa* Ku) were grown in the fruit germplasm repository of Guizhou University, Guiyang, China (26°42.408′ N, 106°67.353′ E) (Figure 1), with a tree age of 4–5 years. Each sample was randomly collected from the base of *R. roxburghii* with a continuous area of 3.35 hectares. Plants showing good growth with mature fruit were randomly selected from different directions of the base. Three fruits were collected from the periphery of the tree crown in four directions of each tree as a biological repeat; each sample had 12 fruits. The samples were snap frozen in liquid nitrogen immediately after collection and stored at −80 °C.

### 2.2. Experimental Methods for Fruit Basic Quality Performance

The single fruit weight was measured by electronic balance (BSA124S, Sartorius, Shenzhen Lintao Instrument Co., Ltd., Shenzhen, China), and the longitudinal and transverse diameters were measured by digital vernier caliper (IP54, Meinaite, Shanghai Meinaite Industrial Co., Ltd., Shanghai, China); the fruit shape index refers to the ratio of longitudinal diameter to transverse diameter of fruit. The titratable acid and soluble solids were measured by sugar acidity meter (PAL-BX|ACID F5, ATAGO CO., Ltd., Tokyo, Japan); the solid acid ratio is the ratio of soluble solids to titratable acid of each fruit. All indexes of each genotype were obtained by taking the average value after measuring the data of each fruit sample.

### 2.3. Sample Preparation and Extraction

Biological samples were freeze dried using a vacuum freeze dryer (Scientz-100F). Then, it was crushed by a mixer mill (MM 400, Retsch) with a zirconia bead for 1.5 min at 30 Hz. Then, 100 mg of lyophilized powder was dissolved in 1.2 mL 70% methanol solution, vortexed 6 times every 30 min (30 s per time) and placed in a refrigerator at 4 °C overnight. Samples were then centrifugated at 12,000 rpm for 10 min, after which extracts were filtrated (SCAA-104, 0.22 μm pore size; ANPEL, Shanghai, China, http://www.anpel.com.cn/, accessed on 27 November 2020).

### 2.4. UPLC Conditions

The sample extracts of 6 *R. roxburghii* genotypes were analyzed using a UPLC-ESI-MS/MS system (UPLC, SHIMADZU Nexera X2, www.shimadzu.com.cn/, accessed on 27 November 2020; MS, Applied Biosystems 4500 Q TRAP, www.appliedbiosystems.com.cn/, accessed on 27 November 2020). The UPLC (column, Agilent SB-C18 (1.8 µm, 2.1 mm × 100 mm)), the solvent A of pure water with 0.1% formic acid, and the solvent B of acetonitrile with 0.1% formic acid form the mobile phase. The sample measurement is carried out according to the gradient procedure and the starting conditions of the procedure are 95% A and 5% B. Within 9 min, a linear gradient to 5% A and 95% B was programed and a composition of 5% A and 95% B was kept for 1 min. Then, a composition of 95% A, 5.0% B was adjusted within 1.10 min and held for 2.9 min. The flow velocity was set as 0.35 mL per minute, the column oven was set to 40 °C, and the injection volume was 4 μL. The ESI-triple quadrupole-linear ion trap (QTRAP)-MS was used to alternately interlink effluents.

### 2.5. ESI-Q TRAP-MS/MS

The triple quadrupole-linear ion trap mass spectrometer (Q TRAP), AB4500 Q TRAP UPLC/MS/MS System is equipped with an ESI Turbo Ion Spray interface, using a positive and negative ion mode; LIT and triple quadrupole (QQQ) scans can be obtained from the system and the system was controlled by Analyst 1.6.3 software (AB Sciex). The operation parameters of ESI source as follows: an ion source turbo spray, source temperature 550 °C, ion spray voltage (IS) 5500 V (positive ion mode)/−4500 V (negative ion mode), ion source gas I, gas II, curtain gas was set at 50, 60, and 25.0 psi, respectively; the collision-activated dissociation was high. Instrument tuning and mass calibration were performed with 10 and 100 μmol/L polypropylene glycol solutions in QQQ and LIT modes, respectively. QQQ scans were acquired as reaction monitoring mode (MRM) experiments with collision gas (nitrogen) set to medium. DP and CE for individual MRM transitions were done with further DP and CE optimization. A specific set of MRM transitions was monitored for each period according to the metabolites eluted within this period.

### 2.6. Metabolite Identification and Quantification

Based on MWDB (metware database), the metabolites of the samples were qualitatively and quantitatively analyzed by mass spectrometry. The isotopic signals, the repetitive signals of K^+^, Na^+^ and NH_4_^+^ ions, and the repetitive signals of fragment ions of other larger molecular weight substances were removed. Each peak with a different color represents one metabolite that was detected. The quantification of metabolites was performed by multiple MRM analysis using triple quadrupole mass spectrometry. In MRM mode, the quadrupole first selected the precursor ions of the target material and eliminated the corresponding ions of other molecular weight materials to eliminate the interference preliminarily; the precursor ions were induced to ionize by the collision chamber and broke to form many fragment ions, and the fragment ions were filtered by QQQ to select a required characteristic fragment ion and eliminate the interference of non-target ions. After obtaining the mass spectrum analysis data of metabolites in different samples, the peak area of all mass spectrum peaks was integrated and the mass spectrum peaks of the same metabolite in different samples were integrated and corrected [18].

### 2.7. Identification of the Key Active Ingredients

All metabolites in *R. roxburghii* from the UPLC-ESI-MS/MS analysis were used to perform a query on the Traditional Chinese Medicine Database and Analysis Platform (TCMSP—Traditional Chinese Medicine Systems Pharmacology Database and Analysis Platform (tcmsp-e.com, accessed on 7 August 2021)) [19] and to further analyze the key active ingredients (KAI) among them. We referred to the previous method and standards to determine which of the detected metabolites are traditional Chinese medicine components and to screen out the KAI [17]. Metabolites identified as the KAI of traditional Chinese medicine shall meet drug likeness (DL) ≥ 0.14 and oral bioavailability (OB) ≥ 5%.

### 2.8. Multivariate Statistical Analysis

To remove the influence of concentration differences on pattern recognition, the logarithm (log10) of the peak area matrix of 6 *R. roxburghii* genotypes were obtained and the Poisson normalization was carried out. Metabolites from 18 samples were used for hierarchical clustering analysis (HCA), principal component analysis (PCA), and orthogonal partial least squares discriminant analysis (OPLS-DA) using R software to study metabolite accession-specific accumulation. Unsupervised PCA was performed by statistics function prcomp within R (www.r-project.org, accessed on 27 November 2020). The data were unit variance scaled before unsupervised PCA. The HCA results of samples and metabolites were presented as heatmaps with dendrograms, while Pearson correlation coefficients (PCC) between samples were calculated by the cor function in R and presented as only heatmaps. Both HCA and PCC were carried out by R package pheatmap. For HCA, normalized signal intensities of metabolites (unit variance scaling) were visualized as a color spectrum. Significantly regulated metabolites between groups were determined by VIP ≥ 1 and absolute Log2FC (fold change) ≥ 1. VIP values were extracted from the OPLS-DA result, which was generated using R package MetaboAnalystR. The data were log transform (log2) and mean centering before OPLS-DA. In order to avoid overfitting, a permutation test was performed. Identified metabolites were annotated using the KEGG Compound database [20] (http://www.kegg.jp/kegg/compound/, accessed on 27 November 2020), and the annotated metabolites were then mapped to the KEGG Pathway database (http://www.kegg.jp/kegg/pathway.html, accessed on 27 November 2020).

### 2.9. Other Experimental Methods

Total phenol acids were determined by Folin–Ciocalteu reagent [21]. Total flavonoid content was analyzed by a spectrophotometric method described by Huang [22]. Vanillin-glacial acetic acid colorimetry was used to determine total triterpenes [23]. The organic acids and lipids were measured according to the method described in the *Principles and Techniques of Plant Physiological Biochemical Experiment* [24]. The contents of L-ascorbic acid, total amino acids, and lignin were determined using Nanjing Jiancheng Bioengineering Institute, Beijing Solarbio Technology Co., Ltd., (Beijing, China) and Suzhou Greis Biotechnology Co., Ltd., (Suzhou, China) kits. In order to determine DPPH radical scavenging activity (DPPH), ABTS cation radical scavenging activity (ABTS), and the ferric ion reducing activity (FRAP) Moure et al., [25], Martinez et al., [26] and Benzie & Strain [27] methods were used with minor modification. Antioxidant activity was expressed as mmol of Trolox equivalents per l of fruits.

## 3. Results

### 3.1. Fruit Basic Quality Performance of Six R. roxburghii Genotypes

The single fruit weight of Rr-5 was significantly larger than Rr-1, Rr-4, Rr-7, and Rr-f (Table 1, all *p* < 0.05). The fruit shape index of Rr-1 was 1.11 and was significantly higher than other genotypes (all *p* < 0.05). The fruit shape index of Rr-3 (0.78), Rr-4 (0.77), and Rr-5 (0.68) were similar; the fruit shapes of the three genotypes were also similar. The fruit shape of Rr-7 (0.84) and Rr-f (0.80) are round, which was consistent with the measured fruit shape index. In addition, Rr-7 had the highest soluble solids content of 14.88%, but soluble solids to acidity ratios were not significantly different between Rr-7 and other genotypes. Thus, we concluded that the six genotypes were similar in sweet and sour flavor.

### 3.2. Overview of the Metabolic Profiles of Six R. roxburghii Genotypes Fruits

To better understand the metabolic profile differences among six *R. roxburghii* genotypes, the widely targeted metabolomics of *R. roxburghii* fruits from Rr-5 and other 5 *R. roxburghii* genotypes were investigated based on the UPLC-ESI-MS/MS system and databases. In total, 723 metabolites were identified (Table S1), including 151 phenolic acids, 95 lipids, 92 flavonoids, 84 amino acids and derivatives, 69 organic acids, 44 tannins, 43 nucleotides and derivatives, 37 terpenoids, 19 lignans and coumarins, 26 alkaloids, and 63 others.

**Figure 2:**
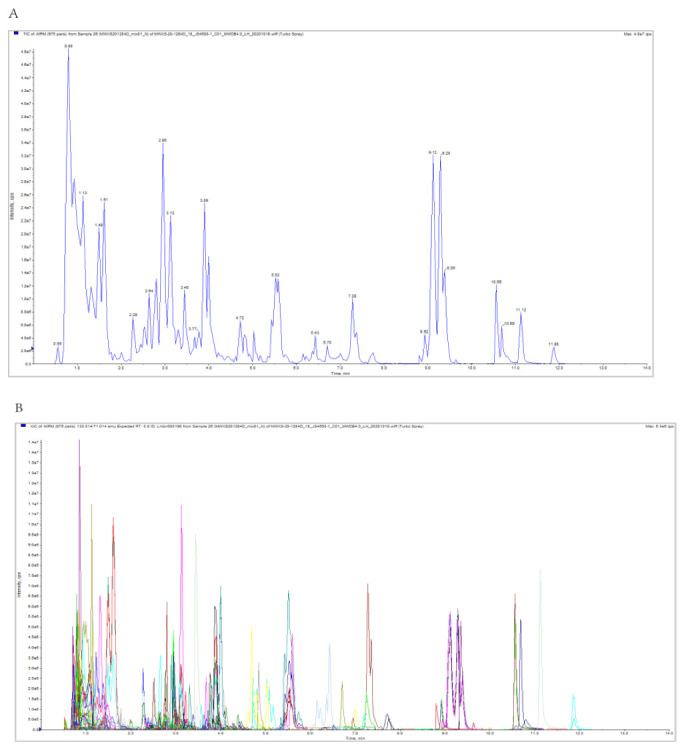
(**A**) TIC of one QC sample by mass spectrometry detection and (**B**) multi-peak detection plot of metabolites in the multiple MRM.

Figure 2A shows a typical total ions current (TIC) plot of one quality control (QC) sample. The mass spectrum data were processed by the Analyst 1.6.3. The figure shows the TIC and MRM metabolite detection multi peak diagram (ion current spectrum extracted by multiple substances, XIC) of mixed QC samples; the abscissa is the retention time (RT) of metabolite detection and the ordinate is the ion current intensity of ion detection (the unit of intensity is CPS, count per second). Based on the local metabolite database, qualitative and quantitative mass spectrometry analyses were conducted on the metabolites in the samples. As shown in Figure 2B, the metabolites of the samples were analyzed qualitatively and quantitatively by mass spectrometry. The multi MRM metabolite detection multi peak diagram in the figure shows the substances that can be detected in the sample. Each mass spectrum peak of a different color represents a metabolite. The characteristic ions of each substance were screened through the triple four-stage rod and the signal strength (CPS) of the characteristic ions was obtained in the detector. The mass spectrometry file of the sample was opened with MultiQuant software to integrate and correct the chromatographic peaks. The peak area of each chromatographic peak represents the relative content of the corresponding substance.

**Figure 3:**
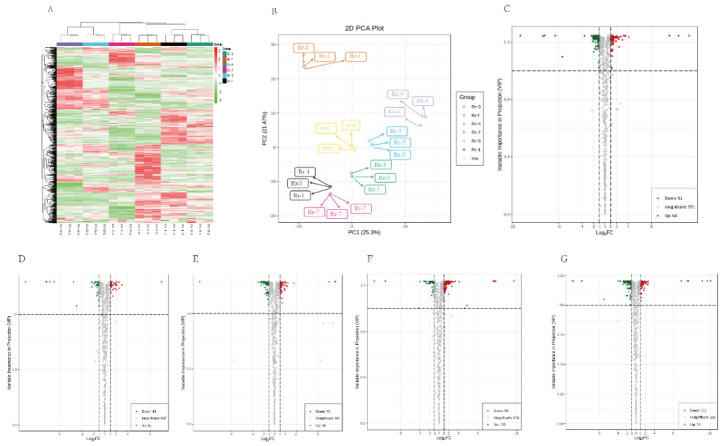
(**A**,**B**) Heat map and PCA of the relative quantification in the detected metabolites among the six *R. roxburghii* genotypes. (**A**) Heatmap for six genotypes. The metabolites of all types were normalized to complete the hierarchical linkage clustering. Red indicates high abundance, green indicates relatively low metabolite abundance. (**B**) Differential metabolites of six genotypes based on PCA. (**C**–**G**) Volcano plots showing up-regulated and down-regulated metabolites between pairs of samples from different genotypes. (**C**) Rr-1 vs. Rr-5. (**D**) Rr-3 vs. Rr-5. (**E**) Rr-4 vs. Rr-5. (**F**) Rr-7 vs. Rr-5. (**G**) Rr-f vs. Rr-5.

Moreover, a cluster analysis of the metabolite profiles of six *R. roxburghii* genotypes was performed (Figure 3A). We observed that metabolic data from the six *R. roxburghii* genotypes were clearly separated. The metabolic profile of Rr-5 contrasted with Rr-7 and Rr-f was varied greatly (Figure 3B). Next, PCA was used to reveal the overall metabolic differences and variability among genotypes. The cumulative contribution rate of the two principal components (PC) reached 49.20%, of which PC1 was 22.75% and PC2 was 26.45% (Figure 3B), and six *R. roxburghii* genotypes were separated in the Figure 3B. According to the degree of aggregation, the Rr-1, Rr-3, and Rr-7, the Rr-4 and Rr-5, and the Rr-f were divided into three categories. In addition, there were also significant differences on the heatmap, indicating that the relative quantification of the metabolites were significantly different among genotypes. This data suggested that six *R. roxburghii* genotypes had distinct metabolic profiles.

### 3.3. Identification of the Key Active Ingredients Belonging to Traditional Chinese Medicines in R. roxburghii

*R. roxburghii* are used as important food sources because of the high content of bioactive substances [1,4,5]. Besides L-ascorbic acid, rutin, quercetin, euscaphic acid, etc., the KAI with health-promoting functions in the *R. roxburghii* have not yet been designated. Therefore, we referred to the previous method and standards to further examine these metabolites in the TCMSP database and to identify the KAI with health-promoting functions for humans in *R. roxburghii*. The results showed that 172 metabolites (Table S2) were the chemical components of traditional Chinese medicine. To further identify the KAI, we refer to previous studies as screening criteria [17]. Fifty metabolites were identified as the KAI in *R. roxburghii*. The 50 metabolites consisted of 12 terpenoids, 8 lipids, 8 phenolic acids, 7 flavonoids, 7 tannins, 4 nucleotides and derivatives, 1 lignan and coumarin, 1 alkaloid, and 2 others. These data suggested that many metabolites in *R. roxburghii*, mainly terpenoids, lipids, flavonoids, phenolic acids, and tannins, have health-promoting functions for humans and the terpenoids, lipids, flavonoids, phenolic acids, and tannins were major contributors.

### 3.4. Differentially Accumulated Metabolites in Fruits of Various Genotypes

A volcano plot was employed to screen the differentially accumulated metabolites (DAMs) of each comparison group. We selected DAMs with two important indicators: a fold change of ≥2 or ≤0.5 and a variable importance in projection (VIP) value of ≥1. VIP values were extracted from the OPLS-DA result (Figure S1). Among them, Rr-5 is the main variety, so this study first compared the DAMs between Rr-5 and other genotypes. As shown in Figure 3, there were 151 DAMs between Rr-1 and Rr-5 (60 were up-regulated), 85 DAMs between Rr-3 and Rr- 5 (41 were up-regulated), 130 DAMs between Rr-4 and Rr-5 (58 were up-regulated), 180 DAMs between Rr-7 and Rr- 5 (130 were up-regulated), and 185 DAMs between Rr-f and Rr-5 (74 were up-regulated). A total of 511 metabolites were different among different genotypes. The results showed that the number of DAMs between Rr-4 and Rr-7 was the highest, followed by Rr-4 and Rr-f, and Rr-7 and Rr-f (Figure S2).

### 3.5. Metabolic Pathway Analysis of DAMs between Rr-5 and the Other Five Genotypes

**Figure 4:**
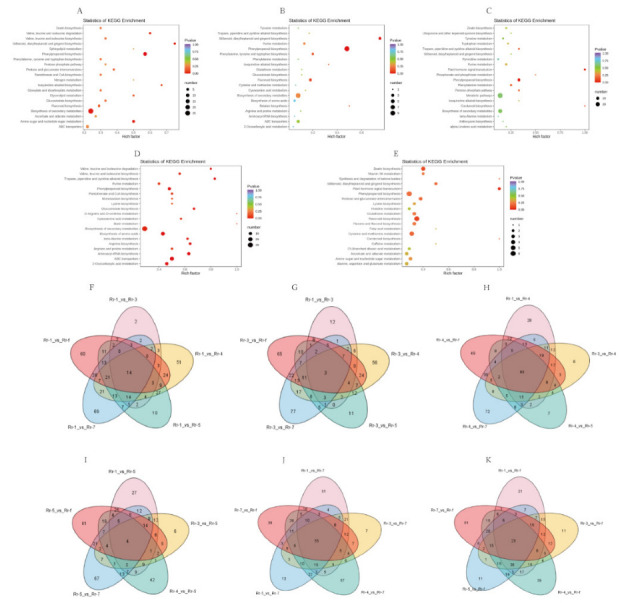
(**A**–**E**) KEGG enrichment analysis of the DAMs between Rr-5 and the other five genotypes. (**A**) Rr-1 vs. Rr-5. (**B**) Rr-3 vs. Rr-5. (**C**) Rr-4 vs. Rr-5. (**D**) Rr-7 vs. Rr-5. (**E**) Rr-f vs. Rr-5. (**F**–**K**) The Venn diagram shows the overlapping and unique DAMs among different genotypes. (**F**) Rr-1 vs. other genotypes. (**G**) Rr-3 vs. other genotypes. (**H**) Rr-4 vs. other genotypes. (**I**) Rr-5 vs. other genotypes. (**J**) Rr-7 vs. other genotypes. (**K**) Rr-f vs. other genotypes.

Identified DAMs (Figure 3C–G) were annotated using the KEGG Compound database and then mapped according to the KEGG Pathway database [17] to obtain detailed pathway information in each comparison group of Rr-5. Seventy-three differential metabolic pathways were observed in between Rr-5 and the other five genotypes (Figure 4A–E). The great majority of metabolites were mapped to “metabolism”, which was consistent with our expectations. In addition, some of the metabolic paths were categorized under “environmental information processing” and “genetic information processing” (Figure S3).

Subsequently, we performed KEGG pathway enrichment analysis to identify the differential metabolic pathways between Rr-5 and the other five genotypes. Forty-six DAMs in the comparison groups of Rr-1 and Rr-5 could be mapped to 59 metabolic pathways in the KEGG database. The relative quantity of 22 DAMs was up-regulated. Enrichment analysis showed that excepted biosynthesis of secondary metabolites, the phenylpropanoid biosynthesis was the most enriched pathway (Figure 4A), and 5 DAMs (2-hydroxycinnamic acid, p-coumaric acid, caffeic acid, ferulic acid, 1-O-sinapoyl-D-glucose) were up-regulated. Comparing Rr-3 and Rr-5, 18 DAMs mapped to 22 metabolic pathways were found; 37 DAMs and 32 metabolic pathways were found after comparing Rr-4 and Rr-5. Interestingly, phenylpropanoid biosynthesis was also the most enriched biological pathway in the two comparison groups (Figure 4B,C).

Figure 4D shows that the biosynthesis of amino acids was the most enriched pathway when comparing Rr-7 and Rr-5, except for the biosynthesis of secondary metabolites, followed by the ABC transporters and arginine biosynthesis. When comparing Rr-f and Rr-5, although the number of metabolites mapped to each metabolic pathway was the highest, the enrichment factor was low (Figure 4E) and among them the most abundant pathway was flavonoid biosynthesis.

### 3.6. Identification of Characteristic Metabolites of Each Genotype

The characteristic metabolites of Rr-1 were determined by multiple comparisons. Fourteen characteristic metabolites were screened (Figure 4F), including ten phenolic acids, two organic acids, and one amino acid. Only the relative quantity of 3,5-O-dicaffeoylquinic acid methyl ester and 3,4- dihydroxy-L-phenylalanine (L-dopa) in Rr-1 were higher compared to other genotypes (Table S3A), which further suggests that 3,5-O-dicaffeoylquinic acid methyl ester and 3,4- dihydroxy-L-phenylalanine (L-dopa) can be used as biomarkers to screen Rr-1.

As shown in Figure 4G, three characteristic metabolites—3,4-dihydroxy- l-phenylalanine (L-dopa), quercetin-5-o-glucuronide, and 2,6-dimethoxyhydroquinone-1-o-glucoside—were confirmed in Rr-3 and only the relative quantity of flavonoids quercetin-5-o-glucuronide was higher compared to other genotypes (Table S3B). Additionally, compared with other genotypes there were fewer characteristic metabolites of Rr-3, which were not concentrated.

The characteristic metabolites of Rr-4, which are shown in Table S3C, mainly included 19 lipids, 9 phenolic acids, and 8 terpenoids. The relative quantities of 3,4-o-dicaffeoylquinic acid methyl ester and 3,5-o-dicaffeoylquinic acid methyl ester in Rr-4 were lower compared to Rr-1, Rr-3, and Rr-5 genotypes and higher compared to Rr-7 and Rr-f genotypes. Moreover, the relative quantities of all detected differentially accumulated lipids (lysoPC 18:1, gingerglycolipid A, lysoPC 19:2, lysoPC 17:1, lysoPC 16:0, lysoPC 18:3, lysoPE 18:1(2n isomer), lysoPE 18:3(2n isomer), lysoPC 18:2) were higher than other genotypes. In addition, most of them were lysophosphatidylcholine isomers, which have a wide range of biological effects and participate in all aspects of the occurrence and development of atherosclerosis.

As shown in Figure 4I, the metabolic profile of Rr-5 was similar to Rr-3. Among the four characteristic metabolites—methyl eugenol, roxburic acid, quercetin-5-o-glucuronide, and hexadienedioic acid—the relative quantity of hexadienedioic acid in Rr-5 was lower than the other genotypes (Table S3D). In addition, the results indicated that the metabolic profile of Rr-5 was similar to the other genotypes.

There were 55 characteristic metabolites identified in Rr-7 (Figure 4J), distributed in ten metabolite categories, except for alkaloids. There were 21 amino acid derivatives and 15 phenolic acids (Table S3E). Among differentially accumulated amino acids, only the relative quantity of l-glutamyl-l-glutamic acid was higher than other genotypes. Also, the relative quantity of 11 phenolic acids (p-coumaroylmalic acid, 4-methoxycinnamic acid, 1-(2,4,5-trimethoxyphenyl)-1,2-propanedione, 3,4-digalloylshikimic acid, syringalide A, 6-O-feruloyl-D-glucose, sinapoyl-p-coumaroyltartaric acid, 1-O-p-coumaroylquinic acid, feruloylferuloyltartaric acid, 4-hydroxybenzaldehyde, p-coumaric acid-4-O-glucoside) was higher than other genotypes. On the contrary, the characteristic metabolites of phenolic acids in Rr-1 were lower.

There were 23 characteristic metabolites in Rr-f (Figure 4K) and these metabolites were evenly distributed in classifications of metabolites. Phenolic acids were the most abundant, followed by organic acids and terpenoids. The relative quantities of methyl caffeate, D-lactulose, senkyunolide K, anthranilate-1-O-sophoroside, 4,8-dihydroxyquinoline-2-carboxylic acid, oxiglutatione, syringaresinol-4′-O-glucoside, (E)-S-(3-(4- hydroxy-3,5- dimethoxyphenyl) allyl) cysteine and 7S,8S-DiHODE, and (9Z,12Z)-(7S,8S)-dihydroxyoctadeca-9,12-dienoic acid were higher compared to other genotypes (Table S3F). Also, the relative quantities of three terpenoids—roxburic acid, 1β, 2α, 3α, 19α, 23-pentahydroxyurs-12-en- 28-oic acid, 27,28-dicarboxyl ursolic acid—were higher than in other genotypes. Therefore, it could be inferred that these metabolites are biomarkers of Rr-f.

### 3.7. Bioactive Substance Content and Antioxidant Capacity of Six R. roxburghii Genotypes

Next, the total phenol, flavonoids, triterpenoids, and ascorbic acid content were examined among different genotypes (Table 2). The ascorbic acid and organic acid of Rr-5 were significantly higher than in Rr-7 and Rr-4 (all *p* < 0.05), respectively; the contents of total phenolic acids and total triterpenes in Rr-7 were significantly higher than in Rr-1, Rr-4, and Rr-5 (all *p* < 0.05). Meanwhile, the FRAP value and ABTS cation radical scavenging activity of Rr-7 were significantly higher compared to Rr-1, Rr-4, and Rr-f (all *p* < 0.05), respectively. Rr-4 had the highest lipid content, but there was no significant difference with other genotypes.

### 3.8. Correlation between Active Substance Content and Antioxidant Capacity

**Figure 5:**
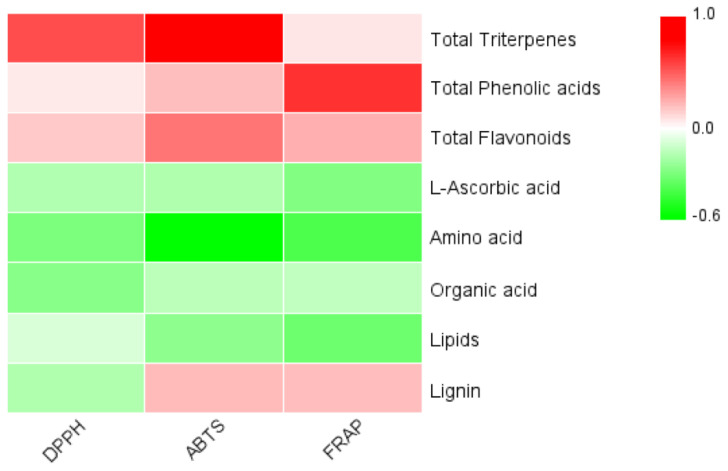
The correlation coefficient between active substances and antioxidant capacity.

As shown in Figure 5, the content of total triterpenoids was positively correlated with DPPH and ABTS (*p* < 0.01) and with FRAP, yet without statistical significance. The content of total phenolic acids was positively correlated with FRAP (*p* < 0.01) and with DPPH and ABTS, yet without statistical significance. Moreover, the content of total flavonoids was positively correlated with ABTS (*p* < 0.05) and amino acids were negatively correlated with ABTS (*p* < 0.05).

The above results showed that phenolic acids, triterpenoids, and flavonoids are significantly related to antioxidant activity, therefore we further analyzed which monomers are significantly related to antioxidant activity. As shown in Figure S4A, among phenolic acid monomers, 4-methoxycinnamic acid, 1-(2,4,5-trimethoxyphenyl)-1,2-propanedione, arbutin, 3,4-digalloylshikimic acid, sinapoyl-p-coumaroyltartaric acid, feruloylferuloyltartaric acid were positively correlated with FRAP and ABTS (*p* < 0.01) and feruloylsinapoyltartaric acid was positively correlated with FRAP and ABTS (*p* < 0.05). 4-hydroxybenzaldehyde, p-coumaroylmalic acid, homovanilloylquinic acid, 1-O-p-coumaroylquinic acid, trihydroxycinnamoylquinic acid were positively correlated with ABTS (*p* < 0.01) and FRAP (*p* < 0.05), 1-O-feruloyl-D-glucose, 1-feruloyl-sn-glycerol, 6-O-feruloyl-D-glucose, 3,4,5-tri-O-galloylshikimic acid, and p-coumaric acid methyl ester were positively correlated with FRAP (*p* < 0.01) and ABTS (*p* < 0.05), 2-feruloyl-sn-glycerol, 1-O-[(E)-p-coumaroyl]-D-glucose, p-coumaric acid-4-O-glucoside, 1-O-feruloylquinic acid, syringalide A were extremely significant positively correlated with ABTS (*p* < 0.01) and 4-nitrophenol, dihydrocaffeic acid, 3-O-p-coumaroylquinic acid-O-glucoside were positively correlated with ABTS (*p* < 0.05). Lancerin was extremely positively correlated with FRAP (*p* < 0.01).

As shown in Figure S4B, among flavonoid monomers, quercetin-3-O-(6″-p-coumaroyl) galactoside, quercetin-3-O-(6″-p-coumaroyl) glucoside was positively correlated with FRAP (*p* < 0.01) and with ABTS (*p* < 0.05). Cyanidin-3-O-glucoside (kuromanin) was positively correlated with ABTS (*p* < 0.01). Phloretin-4′-O-glucoside (trilobatin), naringenin-7-O-neohesperidoside (naringin) were positively correlated with ABTS (*p* < 0.05). Nobiletin (5,6,7,8,3′,4′-hexamethoxyflavone) and cyanidin-3-O-glucoside (kuromanin) were positively correlated with DPPH (*p* < 0.05).

As shown in Figure S4C, among triterpenoid monomers, corosolic acid methyl ester and madecassic acid were positively correlated with FRAP (*p* < 0.01) and with ABTS (*p* < 0.05). Pomolic acid was positively correlated with FRAP (*p* < 0.01). Camaldulenic acid and euscaphic acid were positively correlated with ABTS (*p* < 0.01) and with FRAP (*p* < 0.05). Elemol was significantly correlated with ABTS and FRAP (*p* < 0.05). δ-Amyrenone, ursolic acid, 3β,19α-dihydroxyolean-12-en-28-oic acid, madasiatic acid, and 2α,3β,23-trihydroxy-12-ene-28-ursolic acid were significantly correlated with FRAP (*p* < 0.05).

## 4. Discussion

Previous studies have been mainly focused on investigating the morphological structure and chemical composition of *R. roxburghii* [28,29,30], the developmental stage of *R. roxburghii* [31], and L-ascorbic acid synthesis of *R. roxburghii* [1], while only a few reported on differences among *R. roxburghii* genotypes [32]. In this study, we focused on the fruit quality and metabolic differences among *R. roxburghii* genotypes. Additionally, we explored the phytochemical characteristics of different *R. roxburghii* genotypes. The fruit quality and metabolic profiles characteristics of six *R. roxburghii* genotypes were deeply and comprehensively explained. This research provides a theoretical reference for the targeted application of *R. roxburghii* in the market.

In this study, ESI-Q TRAP-MS/MS was first applied to identify metabolites in different *R. roxburghii* genotypes. In a previous study, GC-MS and UFLC/Q-TOF-MS were used to analyze and compare the essential oils and constituents in *R. roxburghii* and *R. sterilis* fruits, identifying 135 volatile compounds and 59 compounds in methanol extracts [4]. The chemical components of *R. roxburghii* seed were also analyzed and identified by ultra-high performance liquid chromatography coupled with UPLC-Q-TOF-MSE combined with UNIFI screening platform. As a result, 36 terpenoids and 19 flavonoids were putatively identified [33]. In addition, for aroma compounds, 41 aroma compounds were found in *R. roxburghii* and 19 different aroma compounds between the *R. roxburghii* and *R. sterilis* fruits [34]. These results indicated that the metabolite might vary among different *R.* *roxburghii* genotypes, which could be due to the different synthesis and decomposition of metabolites in different genotypes.

Compared with previous studies, our results identified more categories of metabolites and contained more metabolites. A total of 723 metabolites were detected in different *R.* *roxburghii* genotypes. Among them, 172 metabolites were found in the TCMSP database and 50 key active substances were identified as KAI with health-promoting functions for humans in *R. roxburghii*. This study also first reported and analyzed metabolites in *R. roxburghii* fruit, which may promote human health.

Among the different comparison groups, the number of DAMs between Rr-4 and Rr-7 is the highest, followed by Rr-4 and Rr-f, and Rr-7 and Rr-f (Figure S2). In comparison groups with Rr-5, the DAMs between Rr-f and Rr-5 were the most abundant. As expected, the difference in metabolism between the other *R.* *roxburghii* genotypes and Rr-f might be due to the spiny phenotype of the other genotypes. Previous studies proved that there are some structural differences between the acicular trichomes on the fruit surface of *R. roxburghii* and the flagelli and glandular trichomes (GTs) of *R. roxburghii f. eseiosa* fruit. Among them, the acicular trichomes of *R. roxburghii* fruit are rich in starch granins, plastids, and cytoplasm; a large number of cytoplasm and plastids indicate that the cells in the trichomes have high metabolic activity and there are eosinophilic granules in addition to starch grains and cytoplasm in the GTs in *R. roxburghii f. eseiosa* fruit [32]. In addition, trichomes can produce a variety of metabolites when resisting chemical stress [35]. Additionally, a variety of secondary metabolites have also been identified in the GTs of different plants; terpenoids are found in the GTs of *Lychnophora reticulata* [36] and flavonoids are also shown in the glandular hairs of *Matricaria chamomilla* [37], which also proves that the inference above might be reasonable.

In the analysis of metabolic pathways, the phenylpropanoid biosynthesis pathway was the most abundant of DAMs, followed by the biosynthesis of amino acids and flavonoid biosynthesis. The phenylalanine, tyrosine, and tryptophan biosynthesis pathway is the upstream pathway of phenylpropanoid biosynthesis, which produces a large number of secondary metabolites through different branch pathways, including syringin, lignin, methyleugenol, etc., and its intermediate products also participate in the biosynthesis of ubiquinone, flavonoids, etc., [20]. Previous studies revealed that activation of the phenylpropane biosynthesis pathway can be beneficial for plant growth, disease resistance, and fruit quality [38]. Moreover, almost all of the DAMs mapped to this metabolic pathway were up-regulated in Rr-5, therefore it was speculated that Rr-5 had better disease resistance and fruit quality. In addition, the single fruit weight of Rr-5 was the largest and significantly higher than Rr-1, Rr-4, Rr-7, and Rr-f. Additionally, the soluble solids to acidity ratio of Rr-5 did not significantly differ compared to other genotypes, indicating a little difference in sour and sweet flavor between Rr-5 and other genotypes. In addition, the content of ascorbic acid in Rr-5 was the highest and significantly higher than Rr-7 (Table 2); this can also explain why Rr-5 is the main cultivated variety.

Interestingly, by comparing one genotype with the other five, there were 49 characteristic metabolites of Rr-4, of which the relative quantification of 19 lipid metabolites was significantly higher than that of other genotypes (Table S3C); still, there was no significant difference in total lipid content among six *R. roxburghii* genotypes (Table 2). Therefore, these 19 lipid metabolites, most of which are lysophosphatidylcholine isomers, have been suggested as biomarkers of Rr-4. Thus, it was concluded that these up-regulated metabolites could be used as biomarkers of Rr-4. This may be because some monomers were up-regulated in Rr-4, while other monomers were down-regulated, therefore the difference in total lipid content was not significant. Lysophosphatidylcholine can destroy plaque stability, increase oxidative stress, interfere with vascular endothelial function, and induce inflammation [39]. At the same time, the total flavonoids content of Rr-4 was significantly lower than in the other five genotypes. Therefore, due to the accumulation of lipid metabolites in Rr-4, patients with the above-related diseases have been recommended to eat as little as possible of fruit of Rr-4. This also shows the necessity of metabolomic analysis of different *R. roxburghii* genotypes fruit.

The content of total amino acids in Rr-3 was significantly higher than in Rr-4, Rr-5, and Rr-7 (Table 2), but only one flavonoid—quercetin-5-O-glucuronide—was up-regulated in Rr-3 compared to other genotypes (Table S3B), which in turn can be considered as a potential biomarker of Rr-3.

In addition, there were 21 amino acid metabolites in the characteristic metabolites of Rr-7, almost all of which were down-regulated. Additionally, most phenolic acid characteristic metabolites were up-regulated, and these 11 up-regulated substances (p-coumaroylmalic acid, 4-methoxycinnamic acid, 1-(2,4,5-trimethoxyphenyl)-1,2-propanedione, 3,4-digalloylshikimic acid, syringalide A, 6-O-feruloyl-D-glucose, sinapoyl-p-coumaroyltartaric acid, 1-O-p-coumaroylquinic acid, feruloylferuloyltartaric acid, 4-hydroxybenzaldehyde, p-coumaric acid-4-O-glucoside) can be used as biomarkers of Rr-7 (Table S3E). In the measured results of bioactive substances and antioxidant capacity, the total phenolic acid content of Rr-7 and Rr-f was also significantly higher than Rr-1, Rr-4, and Rr-5. The triterpenoids content of Rr-7 was significantly higher than Rr-1, Rr-4, Rr-5, and Rr-f, while the FRAP of Rr-7 was significantly higher than Rr-1, while ABTS of Rr-7 was significantly higher than Rr-1, Rr-4, and Rr-f (Table 2). These results are consistent with the results of correlation analysis, which indicated that the total phenolic acid content and FRAP, the triterpenoids content and DPPH, and ABTS were significantly correlated (Figure 5), which was in line with the previous research results. The correlation analysis results of bioactive substance content and antioxidant capacity of *R. roxburghii* fruit and leaves at different development stages show that phenolic acids have a strong significant correlation with the above three antioxidant capacities, while flavonoids and triterpenes also have a certain correlation [5].

Knowing that the bioactive substances related to the antioxidant capacity of *R. roxburghii* are phenolic acids, triterpenoids, and flavonoids, we further analyzed the correlation between these three categories of metabolite monomers and antioxidant activity. The results showed that 11 triterpene metabolites, 7 flavone metabolites, and 26 phenolic acid metabolites (4-methoxycinnamic acid, 1-(2,4,5-trimethoxyphenyl)-1,2-propanedione, arbutin, 3,4-digalloylshikimic acid, sinapoyl-p-coumaroyltartaric acid, feruloylsinapoyltartaric acid, feruloylferuloyltartaric acid, 4-hydroxybenzaldehyde, p-coumaroylmalic acid, homovanilloylquinic acid, 1-O-p-coumaroylquinic acid, trihydroxycinnamoylquinic acid, 1-O-feruloyl-D-glucose, 1-feruloyl-sn-glycerol, 6-O-feruloyl-D-glucose, 3,4,5-tri-O-galloylshikimic acid, p-coumaric acid methyl ester, 2-feruloyl-sn-glycerol, 1-O-[(E)-p-coumaroyl]-D-glucose, p-coumaric acid-4-O-glucoside, 1-O-feruloylquinic acid, syringalide A, 4-nitrophenol, dihydrocaffeic acid, 3-O-p-coumaroylquinic acid-O-glucoside, lancerin) were significantly correlated with antioxidant activity, including the biomarkers of Rr-7. Therefore, we concluded that the above metabolites have a major role in the antioxidant capacity of *R. roxburghii* fruit. Previous studies have shown that plant-based products rich in natural antioxidants can be used as free radical scavengers to prevent reactive oxygen species (ROS) from damaging healthy cells [40] and to treat diabetes, cancer, cardiovascular disease, neurodegenerative diseases, and inflammatory diseases [12,41]. Based on our data, we concluded that Rr-7 is more suitable for developing natural antioxidant materials.

Although the total triterpenoids content of Rr-7 was significantly higher than that of Rr-1, Rr-4, Rr-5, and Rr-f (Table 2), among the monomers measured in the metabolome Rr-4 and Rr-f had the more terpenoids characteristic metabolites, almost all of which were down-regulated in Rr-4, while roxburic acid, 1β,2α,3α,19α,23-pentahydroxyurs-12-en- 28-oic acid, 27,28-dicarboxyl ursolic acid were up-regulated in Rr-f (Table S3F) that are also biomarkers of Rr-f (1β,2α,3α,19α,23-pentahydroxyurs-12-en- 28-oic acid, 27,28-dicarboxyl ursolic acid were identified in *R. roxburghii* for the first time). Previously, 11 triterpenoids were detected in *R. roxburghii* fruits by UFLC/Q-TOF-MS [4], while in this study 37 terpenoid metabolites were detected, which further advanced the understanding of the metabolic spectrum of *R. roxburghii*. Triterpenoids are also one of the bioactive components in *R. roxburghii* fruit. They are involved in a variety of physiological activities include tumor inhibition, anti-inflammatory, antibacterial, and anti-aging, etc., [11,12,42]. In addition, studies have shown that *R. roxburghii* can relieve the effect of alcohol and slow down the damage of excessive drinking to the liver, because its triterpenoids can affect the oxidative defense system and improve superoxide dismutase in the liver by activating the keap1-Nrf2 signal pathway [43]. Almost all the terpenoid monomers identified in the above studies were identified in this study. While many previous studies have detected roxburic acid, in the present study it was also identified as one of the biomarkers of Rr-f; therefore, it could be inferred that Rr-7 and Rr-f might have higher health-promotion effects than other genotypes.

## 5. Conclusions

In the study, we used the UPLC-ESI-MS/MS system to carry out widely targeted metabolomics research on six *R. roxburghii* genotypes. At the same time, the fruit quality and bioactive substances content of six genotypes were determined and the correlation between bioactive substances, metabolite monomers, and antioxidant activity was analyzed. The main findings of this study are the following: First, we found certain differences in fruit quality and bioactive substances among different *R. roxburghii* genotypes. Second, the possible reasons why Rr-5 was used as the mainly planted variety were obtained. Third, we found new biomarkers for various genotypes and suggested that Rr-7 should be used more in products related to antioxidant activity and that Rr-f should be used in products with health promotion functions. Finally, we used correlation analysis to identify the metabolites with a major role in the antioxidant capacity of *R. roxburghii*. This work will contribute to research overall and a deep understanding on metabolite profiles in *R. roxburghii*. Our results provide the theoretical basis for the application of six *R. roxburghii* genotypes in the food processing industry and medicine. Most importantly, the research provides a specific direction for the breeding of *R. roxburghii*.

## Figures and Tables

**Table 1:** Fruit basic quality performance of six *R. roxburghii* genotypes (Lowercase letters in the table indicate significance, and different letters indicate significant differences among the groups).

	Single Fruit Weight (g)	Longitudinal Diameter (cm)	Transverse Diameter (cm)	Shape Index	Titratable Acidity (%)	Soluble Solids (%)	Soluble Solids to Acidity Ratio
Rr-1	17.22 ± 1.03 c	3.69 ± 0.11 a	3.38 ± 0.13 b	1.11 ± 0.05 a	1.81 ± 0.13 a	12.30 ± 0.41 b	7.08 ± 0.45 a
Rr-3	25.50 ± 1.78 ab	3.39 ± 0.11 ab	4.37 ± 0.07 a	0.78 ± 0.04 b	1.58 ± 0.19 a	11.73 ± 0.44 b	7.66 ± 0.94 a
Rr-4	23.65 ± 2.17 b	3.15 ± 0.13 bc	4.12 ± 0.19 a	0.77 ± 0.02 b	1.52 ± 0.10 a	11.30 ± 0.65 b	7.45 ± 0.28 a
Rr-5	29.48 ± 1.12 a	3.16 ± 0.03 bc	4.64 ± 0.09 a	0.68 ± 0.01 b	1.81 ± 0.23 a	10.30 ± 0.25 b	5.99 ± 0.66 a
Rr-7	13.94 ± 0.90 c	2.78 ± 0.13 cd	3.27 ± 0.06 b	0.84 ± 0.03 b	2.02 ± 0.23 a	14.88 ± 0.55 a	7.68 ± 0.98 a
Rr-f	13.89 ± 1.09 c	2.58 ± 0.07 d	3.21 ± 0.09 b	0.80 ± 0.01 b	2.17 ± 0.22 a	11.48 ± 0.85 b	5.44 ± 0.56 a

**Table 2:** The L-ascorbic acid, total flavonoids, total phenolic acids, triterpenoids, amino acid, organic acid, lignin, lipids content, and the DPPH, ABTS, FRAP in all the samples. (Lowercase letters in the table indicate significance, and different letters indicate significant differences among the groups).

	L-Ascorbic Acid (mg/g Protein)	Total Flavonoids (mg/100 g)	Total Phenolic Acids (mg/100 g)	Total Triterpenes (mg/100 g)	Amino Acid (umol/g)	Organic Acid (%)	Lignin (mg/100 g)	Lipids (%)	DPPH (mmol/L)	FRAP (mmol/L)	ABTS (mmol/L)
Rr-1	851.96 ± 112.27 ab	958.92 ± 78.85 a	1190.15 ± 109.68 bc	3062.30 ± 195.02 bc	89.64 ± 2.63 ab	3.28 ± 0.14 ab	2063.67 ± 153.34 bc	1.50 ± 0.27 a	8.84 ± 0.06 a	2.39 ± 0.30 b	4.45 ± 0.30 c
Rr-3	924.91 ± 117.64 ab	1025.16 ± 32.72 a	1558.38 ± 169.39 ab	3306.17 ± 111.23 ab	108.85 ± 6.56 a	3.49 ± 0.32 ab	1900.04 ± 253.65 c	1.45 ± 0.32 a	9.26 ± 0.27 a	4.45 ± 0.45 ab	5.25 ± 0.20 abc
Rr-4	1044.99 ± 123.88 ab	509.82 ± 58.35 b	857.48 ± 118.01 c	2281.02 ± 262.28 cd	88.08 ± 4.77 b	2.79 ± 0.23 b	2213.40 ± 270.76 abc	1.56 ± 0.60 a	8.75 ± 0.16 a	4.62 ± 0.63 ab	4.46 ± 0.08 c
Rr-5	1209.30 ± 129.95 a	1141.13 ± 84.53 a	1152.05 ± 122.29 bc	3209.38 ± 297.73 b	46.81 ± 4.65 c	3.74 ± 0.29 a	2962.14 ± 252.28 a	1.51 ± 0.53 a	8.77 ± 0.23 a	5.05 ± 0.77 ab	5.32 ± 0.36 ab
Rr-7	672.14 ± 38.60 b	1172.26 ± 53.10 a	1945.55 ± 57.68 a	4014.20 ± 199.50 a	43.43 ± 2.81 c	3.16 ± 0.01 ab	2683.00 ± 180.11 ab	1.30 ± 0.41 a	9.32 ± 0.22 a	8.17 ± 2.08 a	6.00 ± 0.22 a
Rr-f	886.82 ± 123.25 ab	1000.72 ± 161.42 a	1731.80 ± 118.41 a	2110.92 ± 247.97 d	104.81 ± 9.9 ab	3.49 ± 0.18 ab	2101.20 ± 111.44 bc	1.16 ± 0.50 a	8.39 ± 0.52 a	6.75 ± 1.22 a	4.55 ± 0.22 bc

## Data Availability

The data supporting the results of this study are included in the present article.

## Supplementary Materials

The following are available online at https://www.mdpi.com/article/10.3390/foods11060850/s1, Figure S1. Differential metabolite analysis on basis of orthogonal partial least squares-discriminant analysis. (A) OPLS-DA model validation diagram for the comparison groups of Rr-1 and Rr-5. (B) Rr-3 and Rr-5. (C) Rr-4 and Rr-5. (D) Rr-7 and Rr-5. (E) Rr-f and Rr-5; Figure S2: analysis and Volcano plots depicting the upregulated and downregulated metabolites between pairs of samples from different genotypes. (A) OPLS-DA model validation diagram and Volcano plots depicting the upregulated and downregulated metabolites for the comparison groups of Rr-1 and Rr-3. (B) Rr-1 and Rr-4. (C) Rr-1 and Rr-7. (D) Rr-1 and Rr-f. (E) Rr-3 and Rr-4. (F) Rr-3 and Rr-7. (G) Rr-3 and Rr-f. (H) Rr-4 and Rr-7. (I) Rr-4 and Rr-f. (J) Rr-7 and Rr-f; Figure S3. Kyoto Encyclopedia of Genes and Genomes (KEGG) classification results. (A)–(E) The differential metabolites KEGG classification of the comparison group Rr-1 versus Rr-5, Rr-3 versus Rr-5, Rr-4 versus Rr-5, Rr-7 versus Rr-5, and Rr-f and Rr-5, respectively; Figure S4. Correlation analysis of phenolic acid, flavonoid, triterpenoid metabolites and antioxidant capacity. (A) Correlation analysis of phenolic acid metabolites and antioxidant capacity. (B) Correlation analysis of flavonoid metabolites and antioxidant capacity. (C) Correlation analysis of triterpenoid metabolites and antioxidant capacity.; Table S1. All metabolites of 6 R. roxburghii genotypes were identified; Table S2. Metabolites were found to be the chemical compositions of Traditional Chinese Medicines; Table S3. The list of characteristic metabolites identified in each genotyoes. (A) The list of characteristic metabolites identified in Rr-1. (B) in Rr-3. (C) in Rr-4. (D) in Rr-5. (E) in Rr-7. (F) in Rr-f.
